# The role of the skin barrier in modulating the effects of common skin microbial species on the inflammation, differentiation and proliferation status of epidermal keratinocytes

**DOI:** 10.1186/1756-0500-6-474

**Published:** 2013-11-18

**Authors:** Patrick Duckney, Heng Kuan Wong, José Serrano, Diaraf Yaradou, Thierry Oddos, Georgios N Stamatas

**Affiliations:** 1 Durham University, School of Biological and Biomedical Sciences, South Road, Durham DH1 3LE, United Kingdom; 2 Johnson & Johnson Santé Beauté France, Campus de Maigremont, Val de Reuil 27100, France; 3 Johnson & Johnson Santé Beauté France, 1 rue Camille Desmoulins, Issy-les-Moulineaux 92787, France

**Keywords:** Microbial infection, Skin barrier, Stratum corneum, Reconstructed human epidermis, Skin microbiome, Keratinocyte differentiation, Inflammation

## Abstract

**Background:**

Skin resident microbial species are often thought of either as pathogenic or commensal. However, little is known about the role of the skin barrier in modulating their potential for causing disease. To investigate this question we measured the effects of three microbial species commonly found on the skin (*Staphylococcus epidermidis*, *Staphylococcus aureus,* and *Propionibacterium acnes*) on a reconstructed human epidermal model by either applying the bacteria on the model surface (intact barrier) or adding them to the culture medium (simulating barrier breach).

**Results:**

When added to the medium, all of the tested species induced inflammatory responses and keratinocyte cell death with species-specific potency. *P. acnes* and *S. epidermidis* induced specific alterations in the expression of keratinocyte differentiation and proliferation markers, suggesting a barrier reparation response. *S. aureus* induced complete keratinocyte cell death. On the contrary, topically applied *S. epidermidis* and *P. acnes* caused no inflammatory response even when tested at high concentrations, while topical *S. aureus* induced a weak reaction. None of the tested species were able to alter the expression of keratinocyte differentiation or expression markers, when applied topically.

**Conclusions:**

We show that the skin barrier prevents the effects of common skin bacteria on epidermal keratinocyte inflammation, differentiation and proliferation and highlight the importance of skin barrier in defending against the pathogenic effects of common skin bacteria.

## Background

In the last few years it has been conclusively demonstrated that the human skin is an important part of the innate immune system, acting as a first barrier against external microbial threats. In response to pathogenic insults, epidermal keratinocytes secrete an array of soluble cytokines and chemokines to initiate an active immune response [1]. A number of innate defensive pathways are activated, including the generation of reactive oxygen bursts [2,3] and the secretion of human β-defensin, cathelicidin and RNase-family antimicrobial peptides [3].

It is becoming increasingly accepted that commensal species of micro-organisms that naturally reside on the surface of the human skin are an integral part of the innate immune system. For example, *Staphylococcus epidermidis,* the most common cutaneous bacteria, secretes antimicrobial phenol-soluble modulins [4], while stimulating secretion of antimicrobial peptides from keratinocytes, providing increased resistance to pathogen infection [5]. Colonization of the skin by the common microflora may assist in training the developing adaptive immune system by providing epitopes against which the immune system can become primed [6,7]. *S. epidermidis* for example, controls cutaneous immune responses by modulating local T-effector cells through the IL-1 pathway, establishing resistance to infection [8]. The compositions of species that colonize the skin differ between skin sites in correlation with the physiological conditions of the skin microenvironment [9]. Furthermore, the composition of the human skin microflora matures following birth and throughout infancy [6] and possibly through puberty, as the human skin matures in terms of sebaceous activity [10], surface water content [11] and skin surface pH [12]. It could be expected that the skin adjusts its physiological conditions to favor colonization by commensal microflora species over pathogenic species during this period.

However, the same commensal microbial species are also implicated in the pathogenesis of cutaneous infections under altered micro-environmental conditions or when able to overcome the host immune systems [13], as well as in proinflammatory diseases such as acne vulgaris [14], atopic dermatitis [15], and psoriasis [16]. Upon penetration of the skin barrier, members of the skin microflora have been known to elicit diseases such as impetigo, cellulitis, and systemic diseases including endocarditis and sepsis [17]. Knowledge of the immunological interactions between the skin keratinocytes and the skin microflora is imperative in understanding the role of the skin microflora in immune protection and pathogenesis, as well as the factors that determine the commensal or pathogenic nature of the skin microbiome.

During the first years of life, the barrier function and microstructure of infant skin continue to mature [11,18]. It is possible that the early colonization of the skin by commensal bacteria and the subsequent maturation of microbial colony compositions [6] may influence the correct development of the epidermis following birth. Microbial-induced effects on keratinocyte differentiation and proliferation have been observed previously: *P. acnes* has been shown to stimulate normal human epidermal keratinocyte (NHEK) proliferation and affect expression of the differentiation markers Filaggrin, β1 Integrin [19], Transglutaminases 1, 3 and 5, and Keratins 1, 10 and 17 [20]. The ability of members of the skin microbiome to modify epidermal keratinocyte properties and the co-maturation of infant epidermal properties with microflora species composition over time may suggest that the skin microflora may be important for skin maturation in early life.

The top layer of human skin, the stratum corneum, presents a physical barrier to microbial migration through the skin. To investigate the importance of the skin barrier in modulating the effects of the skin microflora on the human epidermis, we applied three species of common skin colonizers to the Reconstructed Human Epidermis (RHE) model. The model consists of stratified human epidermal keratinocytes, complete with stratum corneum, making it relevant to test the interactions of microorganisms residing topically or that have penetrated the skin barrier. In this report we investigated the effects of topical and subcutaneous *S. epidermidis*, *S. aureus* and *P. acnes,* on the elicitation of keratinocyte inflammatory responses and the differentiation and proliferation of the RHE keratinocytes. We demonstrate the effects of species-specific responses and discuss how epidermal keratinocytes are impacted by and respond to the skin microflora as resident inhabitants of the skin surface and as opportunistic pathogens when the barrier is breached.

## Results

### Effects of bacteria added in the medium (breached barrier model) on keratinocyte inflammation and viability

**Figure 1:**
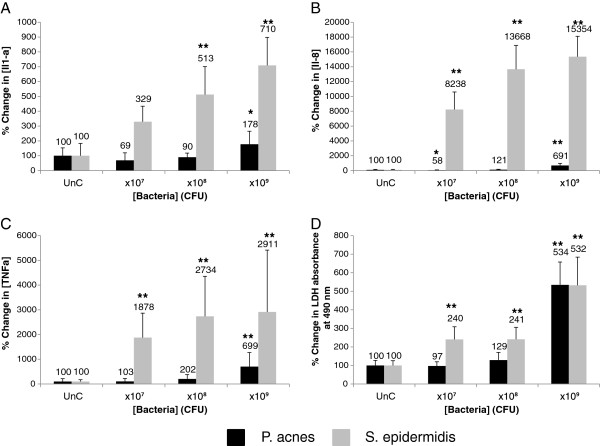
***P. acnes *and *S. epidermidis *added to the medium of reconstructed human epidermal equivalents induce dose-dependent proinflammatory and cytotoxic reactions.** After 24 hrs incubation with bacteria at 1 × 10^7^, 1 × 10^8^ and 1 × 10^9^ CFU, the interleukin (IL)-1α **(A)**, IL-8 **(B)** and TNFα **(C)** levels in the RHE growth medium were measured. The cell viability **(D)** was assessed by measuring leakage of cytoplasmic Lactate Dehydrogenase (LDH) into the RHE growth medium. Data are expressed as percentage of untreated control (UnC = 100%) and are presented as mean ± one standard deviation. Single and double asterisks indicate statistically significant differences (P < 0.05 and P < 0.01) versus the untreated control.

**Figure 2:**
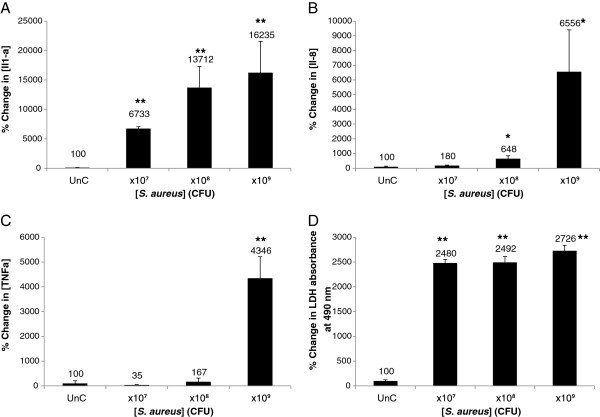
***S. aureus *added to the medium of reconstructed human epidermal equivalents induces strong proinflammatory and cytotoxic reactions.** After 24 hrs incubation with bacteria at 1 × 10^7^, 1 × 10^8^ and 1 × 10^9^ CFU, the interleukin (IL)-1α **(A)**, IL-8 **(B)** and TNFα **(C)** levels in the RHE growth medium were measured. The cell viability **(D)** was assessed by measuring leakage of cytoplasmic Lactate Dehydrogenase (LDH) into the RHE growth medium. Data are expressed as percentage of untreated control (UnC = 100%) and are presented as mean ± one standard deviation. Single and double asterisks indicate statistically significant differences (P < 0.05 and P < 0.01) versus the untreated control.

We tested the effects of bacteria added to the RHE medium, essentially underneath the RHE keratinocytes in order to mimic a scenario in which the bacteria had penetrated the epidermal barrier. Following treatment, the RHE culture medium was screened for pro-inflammatory cytokines and LDH as previously described. All of the tested species induced significant increases in the release of pro-inflammatory cytokines from the RHE keratinocytes and LDH (Figures 1, 2), indicating a pro-inflammatory and cytotoxic effect of the bacteria upon breaching of the epidermal barrier. We observed differential inflammatory and cytotoxic potency between species: *P. acnes* for example was least potent, inducing statistically significant increases in pro-inflammatory cytokine and LDH release only at the highest concentration tested. *S. epidermidis* had a greater pro-inflammatory and cytotoxic effect upon the RHE keratinocytes than *P. acnes*, inducing greater relative increases in cytokine and LDH release at each tested concentration, and induced statistically significant increases in cytokine and LDH release at lower concentrations than *P. acnes* (Figure 1). *S. aureus* was the most cytotoxic and pro-inflammatory of the species added to the RHE medium and stimulated a large and statistically significant increase in IL-1α and LDH release when added at just 1 × 10^7^ CFU (Figure 2). Higher concentrations of *S. aureus* did not elicit greater increases in LDH, suggesting that maximal RHE keratinocyte cell death had been induced. *S. aureus* was even able to stimulate statistically significant increases in IL-1α and LDH release when added to the RHE medium at 1 × 10^2^ CFU (data not shown), showing that it is highly cytotoxic. All RHEs treated with *S. aureus* detached from their basement filters, indicating total keratinocyte cell death. It is likely that the cytotoxicity of *S. aureus* in the medium induced cell death, causing the release of stored IL-1α before synthesis of IL-8 and TNFα could occur and the levels of IL-8 and TNFα released remained unaltered by *S. aureus* in the medium.

### Effects of bacteria added in the medium (breached barrier model) on keratinocyte differentiation and proliferation status

**Figure 3:**
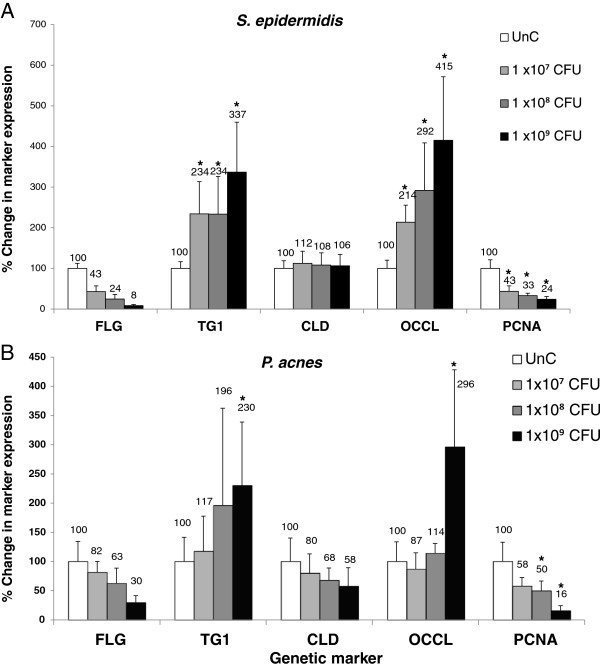
**(A) *S. epidermidis *and (B) *P. acnes *added to the medium of reconstructed human epidermal equivalents induce dose-dependent changes in the expression of genes involved in differentiation (FLG, TG1, CLD, OCCL) and proliferation (PCNA).** After 24 hrs incubation with bacteria at 1 × 10^7^, 1 × 10^8^ and 1 × 10^9^ CFU, RNA from the RHEs was extracted and reverse transcribed. The mRNA expression levels of a range of genes were determined by quantitative reverse transcription polymerase chain reaction. The amount of target transcripts was normalized using an 18S RNA normalization gene. Data are expressed as percentage of untreated control (UnC = 100%) and are presented as mean ± one standard deviation. Asterisks indicate statistically significant differences (P < 0.05) vs the untreated control.

We studied the effects of bacteria added to the RHE medium on the keratinocyte expression of the genetic differentiation and proliferation markers: FLG, TG1, OCCL, CLD and PCNA. Addition of *S. aureus* in the medium induced keratinocyte cell death and degradation of marker mRNA, making it impossible to collect useful data. However, addition of *P. acnes* and *S. epidermidis* induced common patterns of changes in gene expression indicating a conserved response of the keratinocytes (Figure 3). In both cases, adding the bacteria to the medium induced decreased FLG and PCNA expression and increased OCCL and TG1 expression. The expression of CLD was not significantly affected by the presence of *S. epidermidis* or *P. acnes* in the RHE growth medium. As observed with the release of proinflammatory cytokines, *S. epidermidis* induced a stronger response than *P. acnes* in the induction of transcriptional changes of the RHE keratinocytes. *S. epidermidis* was able to induce significant changes in the expression of FLG, TG1, OCCL and PCNA when tested at only 1 × 10^7^ CFU, whereas *P. acnes* was only able to induce significant increases in the affected differentiation markers at 1 × 10^9^ CFU and PCNA at 1 × 10^8^ CFU.

### Effects of topically applied bacteria (intact barrier model) on keratinocyte inflammation and viability

We then tested the potential of topically applied *S. epidermidis*, *P. acnes,* and *S. aureus* to induce proinflammatory cytokine release and cell death in human epidermal keratinocytes. Twenty four hours after treatment of RHEs with topically applied bacteria, RHE culture medium was taken and levels of the proinflammatory cytokines IL-1α, IL-6, IL-8, and TNFα, as well as LDH were quantified to measure epidermal inflammation and cell death respectively. We observed that *P. acnes* and *S. epidermidis* had no impact on the inflammatory status of human epidermal keratinocytes when applied topically and induced little change in IL-1α, IL-8 and TNFα release (Table 1). Topical *S. epidermidis* at 1 × 10^9^ CFU induced a statistically significant increase in IL-8 release compared to the untreated series (P = 0.05). However, the magnitude of this increase could be considered negligible and was elicited only at high bacterial concentrations. Neither species appeared to have cytotoxic effects on the RHE keratinocytes, based on the levels of LDH recovered from the RHE culture medium. *S. aureus* stimulated release IL-1α from the RHE keratinocytes when topically applied only at high concentrations (1 × 10^8^ and 1 × 10^9^ CFU). This was observed in some RHE replicates, but not others, accounting for the high variability observed in the data. Coinciding with this, we also observed large increases in LDH release stimulated in these same replicates. It is likely that topically applied *S. aureus* at 1 × 10^8^ and 1 × 10^9^ CFU induced keratinocyte cell death and subsequent release of stored IL-1α [21], before synthesis of IL-8 and TNFα could occur. No IL-6 was detected in the growth medium of the untreated or treated series.

**Table 1:** **Average release of the proinflammatory cytokines IL-1α, IL-8, TNFα (pg/ml) and LDH (measured by optical absorbance at 490 nm) from RHE 24 hours after topical treatment with *S. epidermidis*, *P. acnes *** **and *S. aureus *** **at different concentrations**

	**Untreated control**	**1×10**^**7 **^**CFU**	**1×10**^**8 **^**CFU**	**1×10**^**9 **^**CFU**
***S. epidermidis***				
[IL-1α] released	100 ± 3.5	116 ± 111.2	143.6 ± 156.8	186.8 ± 215.8
[IL-8] released	100 ± 18.4	151.7 ± 248.8	118.9 ± 156	193.4 ± 239.5*
[TNFα] released	100 ± 0.6	124.4 ± 35.6	138 ± 77.1	151.8 ± 80.7
LDH absorbance	100 ± 0.03	83.6 ± 63.2	82.6 ± 62.9	88.6 ± 58.2
***P. acnes***				
[IL-1α] released	100 ± 8.28	123.1 ± 100.3	122 ± 91	133.4 ± 112.1
[IL-8] released	100 ± 42.08	120.7 ± 129.6	109.5 ± 97.3	107.1 ± 84.9
[TNFα] released	100 ± 1.6	162.6 ± 158.1	115.7 ± 126.1	88.6 ± 113.6
LDH absorbance	100 ± 0.04	100.2 ± 148.4	108.8 ± 161.6	102.1 ± 142.8
***S. aureus***				
[IL-1α] released	100 ± 2.3	157.6 ± 303.8	1062.1 ± 3556.2	1251.7 ± 3735.6
[IL-8] released	100 ± 12.1	60.4 ± 27.7	93.1 ± 95.1	73 ± 2.6
LDH absorbance	100 ± 0.1	273.3 ± 357.5	505.2 ± 1002	666.4 ± 1321

Results are expressed as percentage of the untreated control.

*Single asterisk indicate statistically significant differences (P < 0.05) versus the untreated control.

### Effects of topically applied bacteria (intact barrier model) on keratinocyte differentiation and proliferation

Finally, we tested whether topically applied *S. epidermidis*, *P. acnes* and *S. aureus* could affect the differentiation and proliferation of RHE keratinocytes. *S. epidermidis* and *P. acnes* were tested at 1 × 10^7^, 1 × 10^8^, and 1 × 10^9^ CFU, while *S. aureus* was tested at 1 × 10^3^, 1 × 10^5^, and 1 × 10^7^ CFU as its cytotoxicity at high concentrations caused degradation of mRNA prohibiting further genetic analysis. The keratinocyte expression of the differentiation markers FLG (Profilaggrin), TG1 (Transglutaminase-1), CLD (Claudin-1) and OCCL (Occludin), as well as the proliferation marker PCNA (Proliferating Cell Nuclear Antigen), were measured following topical treatment with the tested species. None of the tested species induced any marked or significant change in the expression of the differentiation or proliferation markers at the tested concentrations (Table 2).

**Table 2:** **Average expression of the RHE differentiation markers FLG, TG1, CLD, OCCL and the proliferation marker PCNA (measured as % expression of untreated control) 24 hours after topical treatment with *S. epidermidis, P. acnes *** **and *S. aureus *** **at different concentrations**

	**Untreated control**	**1×10**^**7 **^**CFU**	**1×10**^**8 **^**CFU**	**1×10**^**9 **^**CFU**
***S. epidermidis***				
FLG	100 ± 21.1	105 ± 50.5	113 ± 60	99 ± 37.4
TG1	100 ± 11	95 ± 30.7	95 ± 33.5	92 ± 25.8
CLD	100 ± 29.2	142 ± 68.3	161 ± 105.2*	123 ± 53.5
OCCL	100 ± 17.2	84 ± 30.9	95 ± 35.4	113 ± 63.8
PCNA	100 ± 25.3	129 ± 36.2	120 ± 39.2	135 ± 34*
***P. acnes***				
FLG	100 ± 28	110 ± 26.5	101 ± 42	99 ± 31.4
TG1	100 ± 26.9	125 ± 74.7	84 ± 29.9	77 ± 34.6
CLD	100 ± 36.5	90 ± 35.7	71 ± 32.1*	84 ± 31.6
OCCL	100 ± 33.4	112 ± 72.9	81 ± 40.5	97 ± 42.6
PCNA	100 ± 18.6	102 ± 49.4	132 ± 108.2	140 ± 93.9
***S. aureus***		**1×10**^**3 **^**CFU**	**1×10**^**5 **^**CFU**	**1×10**^**7 **^**CFU**
FLG	100 ± 19.3	121 ± 32.6	107 ± 20.5	141 ± 45.9
TG1	100 ± 12	179 ± 65.8	112 ± 31.7	114 ± 34.3
CLD	100 ± 22.4	107 ± 25.3	126 ± 30.2	141 ± 43.8*
OCCL	100 ± 31.9	139 ± 67.7	141 ± 43.9	143 ± 45.7*
PCNA	100 ± 18	104 ± 22.6	98 ± 14.3	97 ± 48

*Single asterisk indicate statistically significant differences (P < 0.05) versus the untreated control.

## Discussion

In this study we investigated whether the physical barrier presented by the stratum corneum is sufficient to inhibit microbial-induced effects on epidermal inflammation, differentiation, and proliferation status. Three bacterial species commonly found on the human skin surface (*P. acnes*, *S. epidermidis* and *S. aureus*) were tested for their effects on human epidermal keratinocytes in a stratified in vitro model. We show that bacteria introduced into the RHE medium (below the epidermal barrier) induced important changes whereas bacteria applied topically had little effect on the RHE keratinocytes.

Topically applied *P. acnes* and *S. epidermidis* did not elicit significant inflammatory responses from the RHE keratinocytes and displayed no cytotoxicity. Both *P. acnes* and *S. epidermidis* can be considered on one hand as commensal species, while on the other as opportunistic pathogens implicated in inflammatory diseases. Our data suggest that *P. acnes* and *S. epidermidis* are unlikely to contribute to the pathogenesis of inflammatory conditions when residing on top of the skin barrier: especially as these species were tested at concentrations orders of magnitude above the concentrations of between 3 and 6 CFU normally expected on healthy skin [22]. *S. aureus* however did display limited proinflammatory and cytotoxic effects in some replicates when tested at high concentrations. However, it is unlikely that concentrations of *S. aureus* would reach these values on top of human skin *in vivo*, unless the bacteria could overpower a weakened immune system. We also note that none of the tested species were able to alter keratinocyte differentiation or proliferation and our data suggest that topically residing skin bacteria cannot influence the development of the human skin. The barrier posed by the stratum corneum of the RHE model is likely to have prevented the contact and interaction of the tested bacteria with the living RHE keratinocytes, and may prevent the topical skin microflora affecting living keratinocytes *in vivo. S. aureus* however, has been shown to be able to circumvent the stratum corneum barrier in the pathogenesis of diseases such as scalded skin syndrome [23], which may account for the inflammatory effects it induced when topically applied to the RHE models.

When added to the RHE growth medium, the tested species had a large impact on the RHE keratinocytes. *P. acnes* and *S. epidermidis* were able to induce large inflammatory responses from the RHE keratinocytes and were highly cytotoxic. These effects were stronger when induced by *S. epidermidis. P. acnes* and *S. epidermidis* are known to cause infection when introduced to the subcutaneous space by prostheses or catheters that penetrate the skin [17]. In these situations, the concentrations of *P. acnes* or *S. epidermidis* underneath the skin barrier could equal those tested in this experiment and cause large-scale inflammation and tissue death *in vivo. S. aureus* induced complete keratinocyte cell death and subsequent IL-1α release even when tested at low concentrations, indicating the highly pathogenic potential of *S. aureus* upon penetration of the skin barrier.

Specific transcriptional changes in keratinocyte marker expression were also induced when the skin bacteria were added to the RHE culture medium. *P. acnes* and S*. epidermidis* induced identical expression profiles in the keratinocyte markers that appeared to indicate an effort to repair the epidermal barrier function. We observed large increases in the expression of TG1 in RHE keratinocytes treated with *P. acnes* and *S. epidermidis* in the medium. Transglutaminase-mediated crosslinking of protein scaffold networks is an important step in epidermal keratinocyte terminal differentiation, which results in the formation of the stratum corneum barrier (reviewed by Eckert et al. [24]) and the upregulation of TG1 suggests an increased effort to improve epidermal barrier function by the keratinocytes. It is therefore surprising that expression of FLG was downregulated by the bacterial treatment: FLG monomer binding to keratin filaments organizes them into bundles, establishing a protein network that forms the cornified envelope and stratum corneum [25]. Recently however, de Koning et al. [26] observed identical transcriptional changes in FLG and TG1 during epidermal barrier repair, corroborating the hypothesis that *P. acnes* and *S. epidermidis* stimulated barrier repair processes in RHE keratinocytes when added to the medium. de Koning et al. [26] speculate that FLG may be involved in epidermal barrier maintenance, as opposed to repair and is subsequently downregulated as focus is shifted to repair. The tight junction protein OCCL was significantly upregulated by the bacterial treatments, which may indicate an effort of the keratinocytes to improve barrier function through tightening of cell-to-cell contacts. However, we did not observe altered expression of the tight junction protein CLD. It may instead be that OCCL is a degradation target for these bacteria and is upregulated in expression to compensate: OCCL has been shown to be degraded during staphylococcal infection previously [27]. We also observed large decreases in the expression of the proliferation marker, PCNA. One might expect the proliferation rate of RHE keratinocytes treated with bacteria to increase in order to mount an amplified immune response. However, it seems that the RHE keratinocytes favored differentiation and barrier repair over regeneration and proliferation. The specific transcriptional changes induced by bacteria introduced below the RHE epidermal barrier were identical between the tested species and matched previously described profiles elicited in response to barrier disruption [26]. It is possible that epidermal keratinocytes recognize the presence of the bacteria as a barrier disruption signal, as opposed to a scenario in which the microflora are able to modulate gene transcription in epidermal keratinocytes.

In this work we modeled epidermal breach by introducing bacteria to the medium of RHE. This is a first approach to understand skin barrier breach. A more realistic approach could be by damaging the barrier e.g. by scratching or tape stripping. These methods would be valuable for an in vivo setting and cannot be applicable and reproducible for the in vitro RHE model. However, the number of measured parameters in vivo would be limited, unless an invasive biopsy would be used with the associated problems (e.g. inflammatory markers will increase due to the invasive nature of the biopsy).

Another potential limitation of the study is that we used ATCC strains that could likely have distanced genetically from actual human skin bacteria. Isolation of microbial strains collected from human skin is going to be tried in the future on this model. However, genetic variability of such strains between hosts or even between body sites of the same host needs also to be considered.

Our data suggest that topically residing bacteria must be able to penetrate the epidermal barrier in order to affect the activities of epidermal keratinocytes. The human skin microflora has been shown to offer protective immunological benefits against pathogens both directly [4] as well as indirectly by interacting with human skin keratinocytes [5,28]. One scenario of this could be seen during wounding in which *S. epidermidis* is granted access to epidermal keratinocytes and can modulate their immune response [28]. However it is hard to envisage the topical skin microflora having these effects if prevented from interacting with the epidermal keratinocytes by the stratum corneum. We mentioned above the possibility that the skin microflora may provide epitopes to prime the immune system against pathogens following birth [6-8] and the possibility that this early colonization could stimulate the maturation of the skin barrier following birth. It is possible that the skin microflora may be able to interact with epidermal keratinocytes during this period and enter the epidermis via hair follicles and other epidermal appendages [7]. The diverse ways by which topically residing commensals may be able to interact with epidermal keratinocytes remain to be explored.

## Conclusions

Based on the observed data, *P. acnes*, *S. epidermidis* and *S. aureus* were unable to modulate the inflammatory, differentiation and proliferation status of RHE keratinocytes when applied topically. When tested on a breached skin barrier model, *P. acnes* and *S. epidermidis* induced strong inflammatory responses from RHE keratinocytes and induced specific changes in the expression of RHE keratinocyte differentiation and proliferation markers, perhaps indicative of a barrier repair response. *S. aureus* induced complete keratinocyte cell death and release of proinflammatory cytokines when tested even at low concentrations. Together, our data show that these species of common skin bacteria are prevented from affecting human epidermal keratinocytes by the stratum corneum when residing topically, but may contribute to the pathogenesis of inflammatory diseases following penetration of the skin immunity barrier. The skin barrier is therefore an important part of the innate immune system’s defence against the pathogenic effects of common skin bacteria.

## Methods

### Preparation of bacteria

*P. acnes* (ATCC strain 6919) from second passage stock at -80°C was plated on tryptic soy agar for 5 days at 37°C in anaerobic conditions to yield third passage bacteria and was repeated to yield fourth passage bacteria. *S. epidermidis* (ATCC strain 12228) and *S. aureus* (ATCC strain 6538) were taken from second passage stock at -80°C and were plated on tryptic soy agar for 48 hrs at 32°C in aerobic conditions to yield third passage bacteria, and again to yield fourth passage bacteria. Each species of bacteria was suspended in both physiological water and RHE growth medium at 1 × 10^9^ Colony Forming Units (CFU), as estimated by the optical density of the solutions, and confirmed by standard plate count.

### Reconstructed human epidermis (RHE)

RHE tissues were purchased from Skinethic, Lyon, France. Upon delivery, RHE inserts were transferred to 1 ml RHE growth medium free of antibiotics and hydrocortisone. Inserts were incubated for 24 hrs at 37°C and 5% CO_2_ in sterile conditions.

For the subcutaneous treatment, RHE inserts were transferred to wells containing 1 ml RHE growth medium containing *P. acnes, S. epidermidis* or *S. aureus* at 1 × 10^7^ CFU, 1 × 10^8^ CFU and 1 × 10^9^ CFU. For the untreated control, RHE inserts were transferred to 1 ml RHE growth medium without bacteria.

For the topical treatment, 20 μl of bacterial solution containing 1 × 10^7^ CFU, 1 × 10^8^ CFU and 1 × 10^9^ CFU of *P. acnes, S. epidermidis* or *S. aureus* in physiological solution was added directly on top of RHE inserts. For the untreated control, 20 μl of physiological solution without bacteria was applied instead. The solution applied as a drop was then spread uniformly on the surface with a sealed end Pasteur pipette.

For *S aureus*, lower concentrations (1 × 10^2^, 1 × 10^3^, 1 × 10^5^ and 1 × 10^7^ CFU) were also tested in the medium as this species induces cell death at high concentrations.

All treated RHEs were incubated for 24 hrs at 37°C and 5% CO_2_ before the RHE medium was collected and frozen at -80°C and RNA was extracted. The represented values are the mean and the standard deviation of at least three to five independent experiments.

### Biochemical assays

Collected RHE growth medium was screened for Interleukin (IL)-1α, IL-6, IL-8 and Tumor Necrosis Factor-α (TNFα) using the Flourokine MAP Human Base Kit A (R&D Systems, Lille, France) and analyzed on the Bioplex-200 (Bio-rad, Marnes-la-Coquette, France).

Keratinocyte cell viability was estimated by measuring leakage of cytoplasmic Lactate Dehydrogenase (LDH) into the RHE growth medium using the TOX7-1KT Lactate Dehydrogenase-based In-Vitro Toxicology Assay Kit (Sigma-Aldrich, Saint Quentin Fallavier, France) with the procedure provided by the manufacturer.

Results were expressed as percentage change in inflammation markers or in LDH absorbance at 490 nm, compared to the untreated control.

### Measuring changes in keratinocyte gene expression

Each RHE epidermal tissue was physically separated from the inert polycarbonate filter and lysed in 400 μl lysis buffer, consisting of 100 parts RLT buffer (from the Fibrous Tissue Mini Kit (50) (Qiagen, Courtaboeuf, France), to one part 2-Mercaptoethanol (Sigma-Aldrich) inside a ceramic bead tube (Ozyme, Saint Quentin en Yvelines, France). The tubes were shaken at 5000 rpm and RNA was extracted from the solutions using the Fibrous Tissue Mini Kit (50) on the automated RNA extraction unit; the Qiacube (Qiagen) which eluted the samples in 60 μl RLT buffer.

Reverse transcription was performed with the High Capacity Reverse Trancription Kit (Applied Biosystems, Courtaboeuf, France) using 2 μl RT Buffer, 0.8 μl dNTP mix, 2 μl 10 x RT random primers and 4.2 μl nuclease free water per 10 μl sample. The RT reaction was performed at 25°C for 10 min 37°C for 2 hrs.

Quantitative Polymerase Chain Reaction (qPCR) was used to screen for the expression of genes filaggrin (FLG), transglutaminase 1 (TG1), occludin (OCCL), claudin (CLD), and Proliferating Cell Nuclear Antigen (PCNA). The specific primers used in the study are shown in Table 3. The expression of these genes was normalized against the expression of the 18S housekeeping gene. For each PCR reaction, 5 μl DNA solution was added to 12.5 μl Power SYBR Green PCR Master Mix (Applied Biosystems), 0.075 μl L-primer solution, 0.075 μl R-primer solution (Sigma-Aldrich) and 7.35 μl nuclease free water. The reactions were cycled in a 96-well PCR plate on the Mx3000p Stratagene, (Agilent, Massy, France). Changes in expression of a genetic marker were deemed significant if found to be half or double that of the untreated control.

**Table 3:** Sense and antisense primer sequences used in qPCR

**Genetic marker**		**Primer**	**Sequence**
18S Ribosomal RNA gene		Forward	5'-TAAGTCCCTGCCCTTTGTACACA-3'
		Reverse	5'-GATCCGAGGGCCTCACTAAAC-3'
Profilaggrin	FLG	Forward	5'-TGGCAAATCATCATCTCAAGTG-3'
		Reverse	5'-CTGTCCTGGCTAACTCTGG-3'
Occludin	OCCL	Forward	5'-CAAGCGGTTTTATCCAGAGTC-3'
		Reverse	5'-AAGTCATCCACAGGCGAAG-3'
Claudin1	CLD1	Forward	5'-AGTGCTTGGAAGACGATGAG-3'
		Reverse	5'-TTGAACGATTCTATTGCCATACC-3'
Transglutaminase 1	TG1	Forward	5'-TGCTGGATGCCTGCTTATAC-3'
		Reverse	5'-TGCCTCGGGAGTAATCACC-3'
Proliferating cell nuclear antigen	PCNA	Forward	5'-AACCTCACCAGTATGTCC-3'
		Reverse	5'-ATCCATCAACTTCATTTCATAG-3’

### Statistical analysis

Comparison of keratinocyte Interleukin release under test conditions versus untreated control were performed using the Student’s *t*-test at 95% significance level.

## Abbreviations

CFU: Colony forming units; CLD: Claudin; dNTP: Deoxyribonucleotide phosphate; FLG: Filaggrin; IL-1α: Interleukin 1-alpha; IL-6: Interleukin-6; IL-8: Interleukin-8; LDH: Lactose dehydrogenase; NHEK: Normal human epidermal keratinocyte; OCCL: Occludin; PCNA: Proliferating cell nuclear antigen; qPCR: Quantitative polymerase chain reaction; RHE: Reconstructed human epidermis; RT: Reverse transcriptase; TG1: Transglutaminase-1; TLR: Toll-like-receptor; TNFα: Tumour necrosis factor alpha.

## Competing interest

The authors are employees of Johnson & Johnson Santé Beauté France.

## Authors’ contributions

The experiments were designed by GS and TO and were carried out by PD, with the exception of the preparation of bacterial solutions which was performed by DY. The project was supervised by JS and HKW. PD prepared the final manuscript, which was checked and approved by all authors.
